# *TSPO* mutations in rats and a human polymorphism impair the rate of steroid synthesis

**DOI:** 10.1042/BCJ20170648

**Published:** 2017-11-21

**Authors:** David R. Owen, Jinjiang Fan, Enrico Campioli, Sathvika Venugopal, Andrew Midzak, Edward Daly, Aline Harlay, Leeyah Issop, Vincenzo Libri, Dimitra Kalogiannopoulou, Eduardo Oliver, Enrique Gallego-Colon, Alessandro Colasanti, Les Huson, Eugenii A. Rabiner, Puvan Suppiah, Charles Essagian, Paul M. Matthews, Vassilios Papadopoulos

**Affiliations:** 1 Division of Experimental Medicine and Centre for Neuroscience, Department of Medicine, Imperial College London, Hammersmith Hospital, London, U.K.; 2 Research Institute of the McGill University Health Centre, 1001 Decarie Blvd., Bloc E, Montreal, Quebec, Canada H4A 3J1; 3 Department of Medicine, McGill University Health Centre, McGill University, 1001 Decarie Blvd., Suite D05-2212, Montreal, Quebec, Canada H4A 3J1; 4 University College London, Institute of Neurology and The National Hospital for Neurology and Neurosurgery, 23 Queen Square, London WC1N 3BG, U.K.; 5 National Institute for Health Research Clinical Research Facility, Hammersmith Hospital, Imperial College London, Du Cane Road, London W12 0NN, U.K.; 6 Imanova Centre for Imaging Science, Hammersmith Hospital, Du Cane Road, London W12 0NN, U.K.; 7 Centre for Neuroimaging Sciences, Institute of Psychiatry, Psychology and Neuroscience, King’s College, London SE5 8AF; 8 Department of Pharmacology and Pharmaceutical Sciences, School of Pharmacy, University of Southern California, Los Angeles, CA 90089, U.S.A.

**Keywords:** adrenal, cholesterol transport, gonads, lipid droplets, steroids, translocator protein

## Abstract

The 18 kDa translocator protein (TSPO) is a ubiquitous conserved outer mitochondrial membrane protein implicated in numerous cell and tissue functions, including steroid hormone biosynthesis, respiration, cell proliferation, and apoptosis. TSPO binds with high affinity to cholesterol and numerous compounds, is expressed at high levels in steroid-synthesizing tissues, and mediates cholesterol import into mitochondria, which is the rate-limiting step in steroid formation. In humans, the rs6971 polymorphism on the *TSPO* gene leads to an amino acid substitution in the fifth transmembrane loop of the protein, which is where the cholesterol-binding domain of TSPO is located, and this polymorphism has been associated with anxiety-related disorders. However, recent knockout mouse models have provided inconsistent conclusions of whether TSPO is directly involved in steroid synthesis. In this report, we show that *TSPO* deletion mutations in rat and its corresponding rs6971 polymorphism in humans alter adrenocorticotropic hormone-induced plasma corticosteroid concentrations. Rat tissues examined show increased cholesteryl ester accumulation, and neurosteroid formation was undetectable in homozygous rats. These results also support a role for TSPO ligands in diseases with steroid-dependent stress and anxiety elements.

## Introduction

Translocator protein (18 kDa, TSPO) is a ubiquitous cholesterol-binding outer mitochondrial membrane protein containing five transmembrane domains. TSPO is expressed at high levels in steroid-synthesizing tissues such as the adrenal cortex. The adrenal cortex is the site of synthesis of glucocorticoid and mineralocorticoid steroid hormones, which are crucial for mammalian development, physiology, stress response, immune function, and brain function [1]. TSPO has been identified as a high-affinity-binding protein for cholesterol and other compounds. TSPO ligands have been developed as potential therapeutic targets for neuropsychiatric and neurological diseases [2]. Although numerous pharmacological studies have implicated a role of TSPO in steroid hormone biosynthesis [1] and quantitative trait loci analysis in mice identified *Tspo* in a loci-regulating lipid level [3], recent data in mice suggest a controversial role of TSPO in this process [4–8]. Because of the discrepancies observed between various genetic models used to study TSPO in the mouse, we investigated the role of TSPO in the rat and humans.

In the rat, we used zinc finger nuclease (ZFN) technology to perform *Tspo*-targeted genome editing and generated two lines, a null mutant lacking TSPO expression and a line expressing a truncated TSPO protein which lacks the fifth transmembrane domain, containing the cholesterol recognition amino acid consensus (CRAC) motif, which plays an essential role in the ability of this protein to bind cholesterol [9]. In humans, the presence of rs6971 polymorphism on the *TSPO* gene, which causes a non-conservative amino acid substitution, Ala147Thr, in the fifth transmembrane domain, results in altered binding affinity of TSPO for specific ligands [10]. TSPO polymorphism (Rs6971; Ala147Thr) has been also proposed to affect the hypothalamic–pituitary–adrenal axis predisposing carriers to psychiatric disorders, e.g. bipolar disorders, depression, and anxiety [11–14], and affects the response of patients to anxiolytic TSPO drug ligands [15,16]. Here, we show that both *Tspo* mutations in rat models lead to accumulation of esterified cholesterol and reduced response in adrenocorticotropic hormone (ACTH) stimulation; and a *TSPO* polymorphism in humans attenuates ACTH-induced corticosteroid levels. Moreover, basal testosterone production was reduced and neurosteroid formation was undetectable in *Tspo* homozygous mutant rats.

## Experimental procedures

### Genome editing of the *Tspo* gene in rats by microinjecting embryos with optimized ZFNs

ZFNs were designed, assembled, and validated using the cell lines and CompoZr® knockout Zinc Finger Nuclease Technology according to the manufacturer's instructions (CKOZFN64723; Sigma–Aldrich, Saint Louis, U.S.A.). Other ZFNs were manufactured using customer-directed design and validated using the same cell line as the CompoZr® Custom Zinc Finger Nucleases (CSTZFN; Sigma–Aldrich, Saint Louis, U.S.A.). The two plasmids encoding ZFNs were validated using the rat C6 cell line and the Surveyor Mutation Detection assay according to the manufacturer's instructions (http://www.transgenomic.com). The corresponding mRNAs were microinjected into fertilized eggs of wild-type (WT) rat (Sprague Dawley® rat from Charles River Canada; Constant, Canada) at the Centre des Recherches du Centre Hospitalier Universitaire de Montreal, which generated the two founder rats, Rat5 and Rat7. Locus-specific PCR was performed to screen the founders using the following primer pairs: CKOZFN-F: 5′-AGAGCATACTCTTGCCGTCG-3′ and CKOZFN-R: 5′-ACTCCTAAAGGGGTTGCAGG-3′; and COMPOZr-1kbF: 5′-CCTGGATATGCTGTGTCCCC-3′ and COMPOZr-1kbR: 5′-TGATGGGTCATTTGTGCCCT-3′. Normal PCRs generated 362 bp for the WT and 273 bp for the mutant, respectively, and 818 bp for WT and 652 bp for the mutant, respectively. The gene deletions were further confirmed using Sanger sequencing at the McGill University and Génome Québec Innovation Centre (Montreal, Canada).

### RT-PCR analysis

Total RNAs of the rat adrenal glands and testis were extracted using TRIzol reagent (Invitrogen, Gaithersburg, U.S.A.). The first-strand cDNAs were synthesized with anchored oligo (dT)_18_ and random hexamer primer using the transcription first-strand cDNA synthesis kit (Roche Applied Science, Indianapolis, U.S.A.). Normal PCRs were performed using rat *Tspo* gene-specific primers (rTspo-R: 5′-CTGGGGCACACTGTATTCG-3′ and rTspo-F: 5′-TAGCTTTAAAGGCCCCATGC-3′). The standard size was 575 bp. The amplicons were sequenced at the McGill University and Génome Québec Innovation Centre to confirm the mRNA mutations.

### Protein quantification and immunoblot analysis

Protein was extracted from WT and mutant rat adrenal glands with T-PER™ tissue protein extraction reagent (Pierce). Tissue samples were homogenized with T-PER reagent on ice for 2 ×  30 s with 15 s interval as per the manufacturer's instruction (Thermo Scientific, Rockford, U.S.A.) centrifuged at 10 000 ***g*** for 5 min to pellet cellular debris, and the protein concentration in each supernatant was quantified using the Bradford method with bovine serum albumin as the standard. An equal amount of protein (20 µg each) was mixed with sample buffer [240 mM Tris–HCl (pH 6.8), 8% SDS, 80% glycerol, 0.1% bromophenol blue, and 2 mM dithiothreitol] and subjected to SDS–PAGE using a 4–20% gradient gel (Invitrogen, Gaithersburg, U.S.A.). Separated proteins were electroblotted onto nitrocellulose membranes, which were then blocked in 5% fat-free milk overnight, and incubated with primary antibody specific for TSPO (1 : 1000) [9]. The TSPO KO was further validated using commercial rabbit polyclonal anti-TSPO Ab (specific for rat TSPO; Abcam, ab154878; Cambridge, U.S.A.). The loading control was reprobed on the same membrane with anti-hypoxanthine phosphoribosyltransferase (HPRT; Abcam, ab10479, Cambridge, U.S.A.). Blots were then incubated with horseradish peroxidase-conjugated secondary antibody (1 : 5000) and developed with enhanced chemiluminescence (Amersham Biosciences, Piscataway, U.S.A.).

### Expression and purification of recombinant TSPO

The pET15b vector containing WT or Ala147/Thr mutant mouse TSPO cDNA was transformed into the *Escherichia coli* BL21(DE3) strain (Novagen, Madison, U.S.A.). Positive colonies were selected and grown briefly in two 10 ml pre-cultures, followed by a 500 ml culture for the production of recombinant protein, which was induced by 1 mM isopropyl-1-thiol-β-d-galactopyranoside during the exponential growth phase. Cells were harvested by centrifugation at 5000 ***g*** for 15 min using wash buffer containing 150 mM NaCl and 50 mM HEPES-Na (pH 7.8). Lysates were obtained by sonication in an ice water bath for 3 ×  30 s with 10-s rest intervals. Inclusion bodies were obtained by centrifugation at 5000 ***g*** for 20 min and resuspended in wash buffer containing 1% SDS. The solution was centrifuged at 15 000 ***g*** to obtain solubilized inclusion bodies containing recombinant mouse WT or mutant TSPO.

The solubilized inclusion bodies were treated with 2 µl of benzonase (25 units per µl) and purified using a 1.2-ml Superflow Ni-NTA resin column (Qiagen, Chatsworth, U.S.A.) as described previously [17]. Briefly, the samples were passed through the prepared resin column and washed with wash buffer containing detergent and 5 mM imidazole. Recombinant mouse WT or mutant TSPO was obtained by eluting with buffer containing 300 mM imidazole.

### Reconstitution of recombinant TSPO into liposomes

A stock solution of dimyristoyl phosphatidylcholine/dimyristoyl phosphatidylethanolamine (DMPC/DMPE, 9/1) (Avanti Polar Lipids, Birmingham, U.S.A.) was prepared and mixed with purified SDS-solubilized recombinant TSPO at a lipid-to-protein ratio of 5 : 1 (w/w) [18]. The obtained ternary complex was stirred for 15 min before adding small quantities of prepared Bio-Beads SM2 at different time points to remove SDS from the sample and induce vesicle formation. This process was monitored spectrophotometrically at 550 nm absorbance [19]. Reconstituted TSPO proteoliposomes were subsequently obtained by removal of Bio-Beads from the sample.

### Electron microscopy

Proteoliposome samples were applied to carbon-coated 200-mesh copper grids, negatively stained with 1% uranyl acetate, and air-dried. Proteoliposomes were observed with a transmission electron microscope (FEI Tecnai 12; FEI) operated at 120 kV, and images were collected with a CCD camera (AMT XR 80 C).

### Radioligand-binding assay and [^3^H]-PK 11195 autoradiography

Reconstituted TSPO proteoliposomes at 1.0–2.0 µg/ml were used to perform PK 11195- and cholesterol-binding assays as described previously [18]. Bound [^3^H]-PK 11195 and [^3^H]-cholesterol were quantified using liquid scintillation spectrometry. *K*_D_ and *B*_max_ values were obtained using saturation isotherm analyses with Prism 4 in GraphPad (La Jolla, CA, U.S.A.).

PK 11195 saturation-binding assays were performed using WT and TSPO-knockout (KO) male and female rat adrenal protein extracts as described previously [20]. Protein levels were measured using the Bradford assay. Aliquots of 15 µg of protein were incubated in the presence of 0.1–15 nM [^3^H]-PK 11195 (PerkinElmer, specific activity 84.0 Ci/mmol). Nonspecific binding was determined using 10 µM of unlabeled PK 11195 ligand (Sigma, St Louis, MO, U.S.A.). Samples were filtered through Whatman GF/B filters (Brandel, Gaithersburg, MD). Radioactivity trapped on the filters was determined using liquid scintillation counting. Specific [^3^H]-PK 11195 binding was analyzed using the iterative nonlinear curve-fitting program and the extra sum-of-squares *F-*test in Prism 5 of GraphPad (La Jolla, CA).

For PK 11195 autoradiographic assays, adrenal sections were incubated with 1.2 nmol/l [^3^H]-PK 11195 (PerkinElmer, Downers Grove, IL, U.S.A.) (specific activity 80.9 Ci/mmol) for 30 min at room temperature (22°C), in 50 mmol/l Tris–HCl (pH 7.4). After incubation, the slides were washed three times for 3 min in ice-cold incubation buffer. Finally, the slides were rinsed once for 30 s with ice-cold distilled water and dried overnight in a vacuum desiccator containing paraformaldehyde (PFA) powder. [^3^H]-PK 11195 binding was analyzed using digital autoradiography with a Beta-Imager 2000 (Biospace Lab, Paris, France).

### Oil Red O staining

Tissue cryosections of both male and female adrenal glands were embedded in optimum cutting temperature medium, and then 6-µm cryosections were prepared at the histology core facility of the Goodman Cancer Research Centre at McGill University. For Oil Red O (ORO) staining, tissue cryosections were washed twice with PBS and fixed with 4% PFA in PBS for 20 min. After removing PFA, lipids were stained with ORO according to the manufacturer's instructions (NovaUltra™ Oil Red O stain kit, IHC World, Ellicott City, U.S.A.). After repeated washing with distilled water, the tissues were counterstained with Mayer's hematoxylin solution for 2 min, rinsed with distilled water, and then mounted with aqueous slide mounting medium for visualization.

### Homology modeling and molecular docking analyses

The putative 3D structure of rat TSPO was predicted via an automated comparative protein modeling server (Swiss-model; http://www.expasy.ch) with the optimized mode using the co-ordinates of the mouse TSPO NMR structure (PDB accession number: 2MGY), which is available from the Brookhaven Protein Database (PDB) [21,22]. Molecular docking analyses were performed using AutoDock Vina [23]. The 3D co-ordinates of cholesterol were obtained from ChemSpider (http://www.chemspider.com). WT rTSPO was docked with cholesterol using whole protein as the grid-docking box, whereas Rat7-mutated TSPO was docked with cholesterol using the CRAC motif region as a smaller grid-docking box using the same input parameters used for WT rTSPO.

### Hormone treatment, blood plasma sample collection, and measurement of circulating steroids

We evaluated the activity of adrenal glands and testes by measuring the levels of circulating corticosterone and testosterone, respectively, in response to acute stimulation with human ACTH and chorionic gonadotropin (hCG). Brain steroid production was assessed by measuring allopregnanolone levels in the cortex. Blood was collected 6 days before euthanasia via submandibular puncture, and plasma was extracted and stored at −80°C until used. Samples were used to determine basal hormone levels before application of hormone challenge. Three days before euthanasia, mice were intraperitoneally injected with 10 IU of hCG (National Hormone and Peptide Program). After 1 h, blood was collected via submandibular puncture, and plasma was separated and stored at −80°C until use. On the day of euthanasia, ACTH fragment 1–24 (Sigma–Aldrich) was administered subcutaneously at a dose of 500 ng/g body weight. After 1 h, rats were killed, and the adrenals and testes were removed and snap-frozen in liquid nitrogen or fixed in 4% PFA. Blood was collected by cardiac puncture; plasma was separated by centrifugation at 2000 ***g*** for 15 min and stored at −80°C until further use. Plasma corticosterone levels were measured using radioimmunoassay as described previously [6]. Enzyme-linked immunosorbent assay (ELISA) was used to measure testosterone (Cayman Chemicals, Ann Arbor, MI, U.S.A.) according to the manufacturer's instructions. Absorbance was measured at 420 nm using the VICTOR™ X5 Multilabel Plate Reader (PerkinElmer, Inc., Waltham, MA, U.S.A.). The identity and levels of measured testosterone and corticosterone in the blood were confirmed by liquid chromatography–mass spectrometry (LC–MS) using an AB Sciex 5600 triple-TOF/MS, time-of-flight mass spectrometer system fitted with a ‘Turbo-V’ ionization source and run in full-scan (50–800 µm), positive-ion mode. Aliquots of plasma were extracted using liquid/liquid extraction with methyl-tertiary butyl ether. Dried extracts were reconstituted in 15 µl of 10% acetonitrile in water. A 10 µl volume was injected into the LC–MS system. Testosterone (*m/z* 289) (general retention time, *t*_R_, 3.50 min) and corticosterone (*m/z* 347) (*t*_R_ 3.07 min) were chromatographically separated with a linear gradient on a Shimadzu Nexera XR HPLC system. The analytical column was a Thermo HyPurity C18 (30 mm × 2.1 mm ID, 3.0 µm) column. A binary liquid mobile phase was utilized consisting of (A) 0.1% formic acid in water and (B) 0.1% formic acid in acetonitrile. The flow rate was 500 µl/min, the injection volume was 10 µl, and the total run time was 10 min.

Allopregnanolone levels in brain cortex homogenates were measured after extraction by ultrahigh performance liquid chromatography (UHPLC)–triple quadrupole mass spectrometry (QQQ) using a Thermo Scientific TSQ Quantiva Triple Quadrupole Mass Spectrometer with a heated electrospray ionization source and a Thermo-Scientific UltiMate™ 3000 UHPLC system (UltiMate™ 3000 RS autosampler with a cooled tray holder). The relative steroid amounts were estimated from reference standards in solution (allopregnanolone and a d4-allopregnanolone as the internal standards at 10.0 ng/ml for each analyte).

### Genotype-dependent response to ACTH treatment in humans

To assess the functional significance of rs6971 (A147T) in humans, we evaluated basal and ACTH-challenged cortisol production in healthy male volunteers aged 18–40 years old. The clinical trial was approved by the Central London REC 4 ethics committee (10/H0715/45). Exclusion criteria used included history of disease affecting the hypothalamic pituitary disease (including mood disorders), recreational or prescription drug use (including contraception), and abnormal sleep patterns. Subjects were genotyped at the rs6971 polymorphism using a Taqman SNP Genotyping Assay (ThermoFisher Scientific, Waltham, MA, U.S.A.), into A147 (WT), A147T [heterozygote (HE)], or T147 (homozygote/mutant). Thirty male subjects were studied, 10 WT (31.1 years, SD 6.5), 10 A147T subjects (25.4 years, SD 6.9), and 10 147T subjects (28.5 years SD 7.1). Subjects were instructed to avoid alcohol and strenuous exercise the day before the study, and avoid all food and drink (but for water) from midnight the night before the study. A venous cannula was placed and 2 ml of blood drawn into tubes containing a rapid clot activator, every 15 min from 09:00 to 11:00 AM inclusive. A commercially available ACTH analog (Synacthen, Mallinckrodt Pharmaceuticals, Staines-Upon-Thames, Surrey, U.K.) was then administered intravenously (250 µg), and blood was drawn in the same way subsequently for a further 90 min. This clinical study design was chosen because of a theoretical risk that the acute anxiety of cannulation with a wide bore needle might alter plasma cortisol, and if so this response could be TSPO genotype-dependent. Hence, we allowed for a 2 h recovery period from cannulation to ACTH administration. Serum cortisol was measured using the Architect system™ delayed one-step immunoassay (Abbott Laboratories, Abbott Park, IL, U.S.A.). Analysis of cortisol concentrations was performed using linear models with gamma errors, as initial exploratory analyses suggested that the usual assumption of normality did not apply to the cortisol concentration data. Tests of trend across genotypes were based on linear contrast coefficients within the linear models (SAS Version 9.3, Cary, NC, U.S.A.).

## Results

### Rat *Tspo* has been knocked out via embryo microinjections of ZFNs

**Figure 1.**
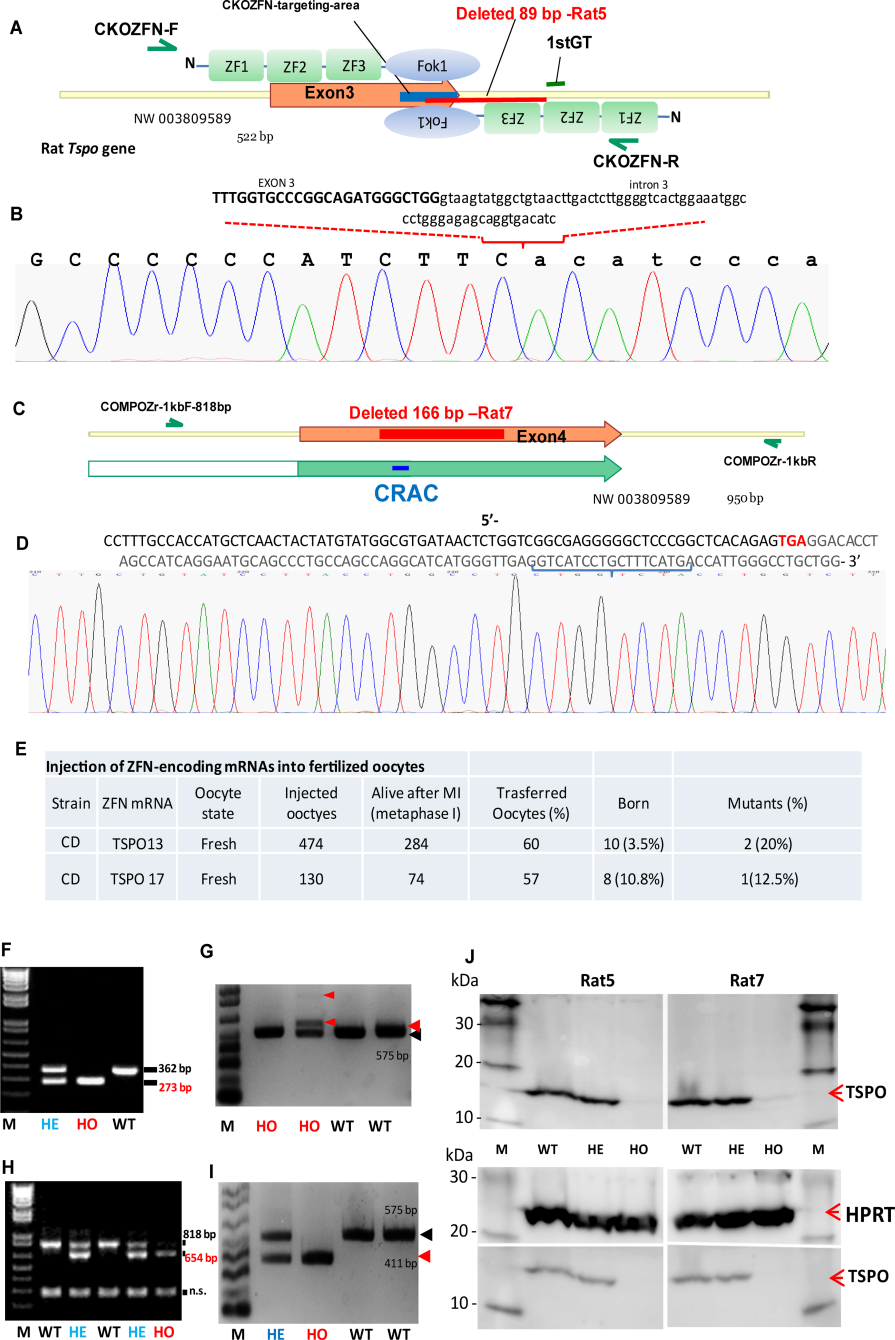
Genome editing of the rat *Tspo* locus using the CompoZr® optimized/customized KO ZFN (CKOZFN) technology. (**A**) A diagram of the ZFN-optimized target region in the rat *Tspo* exon 3 region. The expected CKOZFN targeting area is indicated as a blue line. Three ZFN domains (ZF1, ZF2, and ZF3) linked to the Fokl nuclease (Fok1) domain on each side of the expected target area are indicated to form a ZFN homodimer binding to DNA. The 89-bp deletion in Rat5 is indicated in red. An intron sequence was introduced into the new mRNA, indicated by the first ‘GT’, and insertions were confirmed by sequencing (Supplementary Figure S1C). The indicated primers (CKOZFN-F and CKOZFN-R) were used for founder identification and genotype screening. (**B**) Sequencing confirmation of rat *Tspo* locus modification from the Rat5 founder. The 96-bp deletion disrupts the exon 3-intron 3 junction; the exon sequence is shown in capital letters, and the intron sequence is shown in lower-case letters. (**C**) Diagram of the CRAC-specific deletion by CompoZr® Custom ZFN technology within rat *Tspo* exon 4. The expected targeted area is indicated in blue as the CRAC; the actual deletion in Rat7 is indicated in red within exon 4 (166 bp were deleted within exon 4). The indicated primers (COMPOZr-1kbF and COMPOZr-1kbR) were used for founder and genotype screening. (**D**) Sequencing analysis confirmed that the rat *Tspo* locus was modified in the Rat7 founder. The 166-bp deletion within exon 4 was expected to generate a truncated exon 4. The TGA stop codon is highlighted in red. (**E**) Summary of the fertilized eggs injected with ZFN mRNAs, transferred at metaphase I, and the rare founders that were obtained. TSPO13, Rat5 with exon 3–intron 3 disruption (two founders obtained) and with a larger deletion (one founder obtained, not shown); TSPO17, Rat7 with a CRAC-specific deletion (one founder obtained). (**F**) Agarose gel image of the typical PCR products used for genotype screening of Rat5 with *Tspo* locus modification. WT, 362 bp; HE, two bands (362/273 bp); HO, 273 bp. (**G**) RT-PCR analysis of Rat5 vs*.* WT rat adrenal glands was used to detect mutated mRNA species. Red arrow, Rat5 mutated *Tspo* mRNA (the intron sequence was introduced and resulted in a transcript 10 bp shorter than WT *Tspo* mRNA or transcripts of increasing length up to the full intron sequence fused with exon 4). The 575-bp WT *Tspo* mRNA is indicated. The corresponding rat genotypes are indicated. (**H**) Agarose gel image of typical PCR products used for genotype screening of Rat7 with a mutated *Tspo* predicted to lack the CRAC domain. WT, 818 bp; HE, 818 bp; HO, 654 bp; n.s., nonspecific band. (**I**) RT-PCR of the Rat7 vs. WT rat adrenal glands was used to detect mutated mRNA species. Red arrow: mutated Rat7 *Tspo* mRNAs with a deletion in exon 4 were 411 bp, whereas WT *Tspo* mRNA species were 575 bp. The corresponding rat genotype (WT, HE, or HO) is indicated. (**J**) Immunoblot analysis of proteins extracted from homogenized adrenal gland tissues of Rat5 (left panel) and Rat7 (right panel). The rat genotype (WT, HE, and HO), TSPO, HPRT (loading control), and the protein ladder (kDa) are indicated.

**Figure 2.**
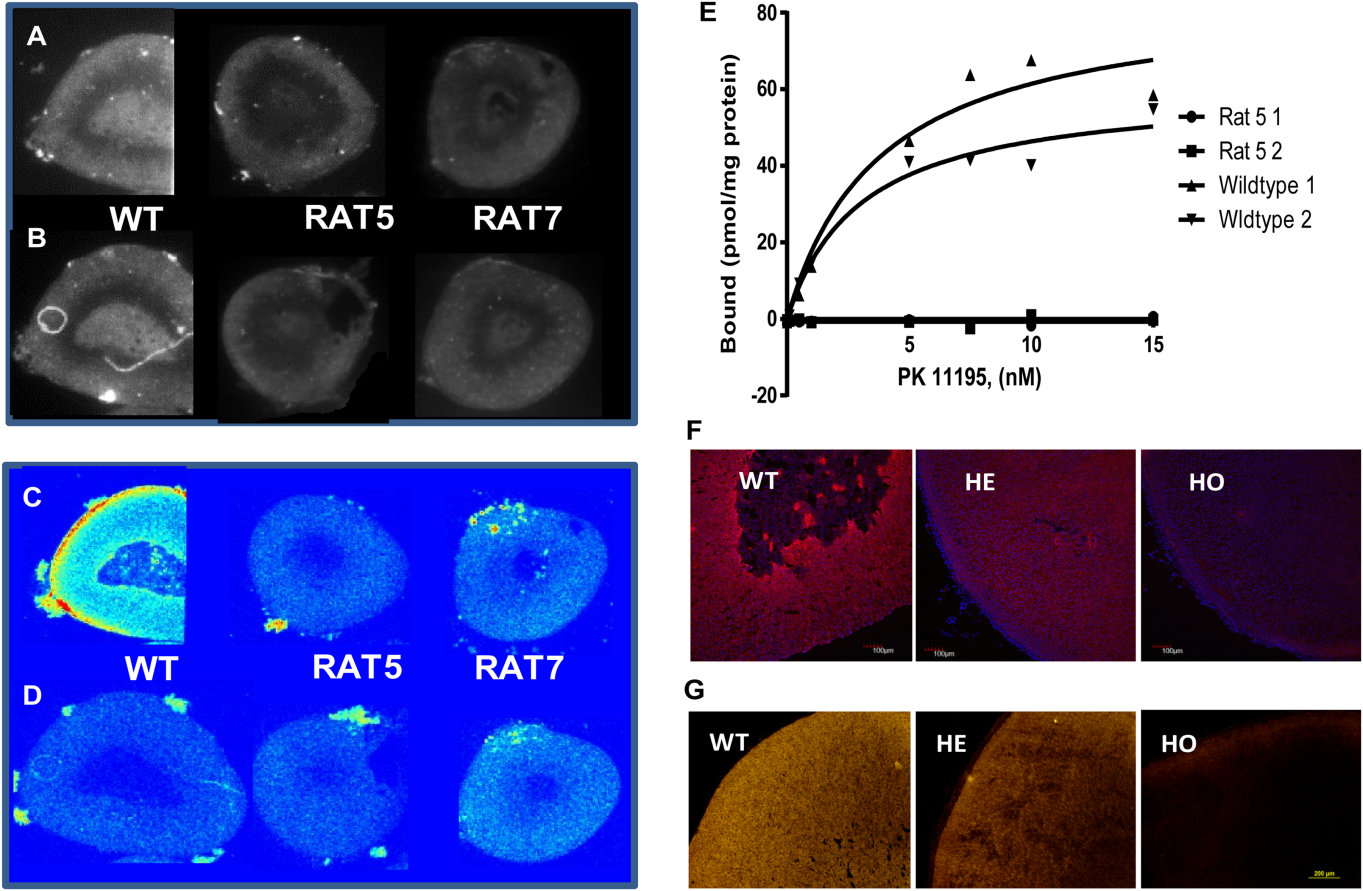
Effect of genome editing of rat *Tspo* on the PK 11195 binding and immunofluorescence staining. (**A** and **B**) Representative optical bright-field images were used as controls to show the morphology of adrenal sections used for the binding assay. (**C** and **D**) Autoradiographic localization of PK 11195 in the adrenal glands from WT, Rat5, and Rat7. (**C**) Autoradiographic images of tissue incubation with 1.2 nmol/l [^3^H]-PK 11195 in 50 mmol/l Tris–HCl (pH 7.4) for 30 min at room temperature (22°C). [^3^H]-PK 11195 binding was analyzed by digital autoradiography using a Beta-Imager 2000 (Biospace Laboratory, Paris, France). Binding intensities are presented in false color using the ImageJ look-up table ‘royal’. (**D**) Autoradiographic images of tissue incubation as in **C** but additionally with cold PK 11195 to show nonspecific binding. (**E**) Saturation isotherm of [^3^H]-PK 11195-specific binding studies using 15 µg of protein extracted from adrenal glands of WT1, WT2, and TSPO KO Rat5-1 and Rat5-2. The *K*_D_ and *B*_max_ of PK 11195 binding to WT1 adrenal protein extract were 3.84 ± 1.62 and 84.95 ± 11.86, respectively, and for WT2, adrenal protein extract was 3.04 ± 0.96 and 60.40 ± 5.67, respectively. (**F** and **G**) IF staining of cryosections of Rat5 adrenal gland using rabbit polyclonal anti-TSPO Ab (NP155). Images were obtained using laser scanning confocal microscopy (**A**) and epifluorescence imaging using an inverted microscope (**B**). The rat genotype (WT, HE, and HO) is indicated. Scale bar = 100 µm.

To assess the role of TSPO *in vivo*, we performed *Tspo*-targeted genome editing in rats by microinjecting embryos with optimized and customized ZFNs designed for targeted gene KO and specific genome editing (Supplementary Figure S1A,B). We generated the following two mutant rat lines: Rat5 with an 89-bp deletion flanking the exon 3/intron 3 junction, and Rat7 with a 164-bp deletion (including Ala147) in the fifth transmembrane loop and the CRAC motif within exon 4 (Figure 1A–D). Approximately 57–60% of the injected fertilized eggs were successfully transferred, but only 3.5% of these resulted in live animals, indicating that the mRNAs and/or KO *Tspo* have a toxic/lethal effect in oocytes (Figure 1E). Rat5 homozygotes (HO) carried an 89-bp deletion (described above), which resulted in *Tspo* mRNA transcripts with varying lengths that were not translated (no TSPO protein expression) (Figure 1F,G,J and Supplementary Figure S1C). Rat7 HO carried a 164-bp deletion within exon 4, which results in truncated mRNA transcripts as observed from RT-PCR analysis, but the anti-TSPO antibody used in immunoblots did not detect the proposed mutant protein (Figure 1H,I,J and Supplementary Figures S1D and S2A,B). Rat5 and Rat7 did not display significantly aberrant phenotypes or exhibit specific binding of the classical TSPO ligand [^3^H]-PK 11195 as assessed by autoradiography (Figure 2A–D and Supplementary Figure S3A–D) and radioligand-binding assays in adrenal tissues (Figure 2E). We further confirmed that no TSPO immunoreactive protein was detected in HO Rat5 adrenal gland using immunofluorescence (IF) analysis using both confocal and epifluorescence imaging (Figure 2F,G).

### Rats carrying *Tspo* deletion mutations show disturbed neutral lipid accumulation and no or reduced response to ACTH treatment

**Figure 3.**
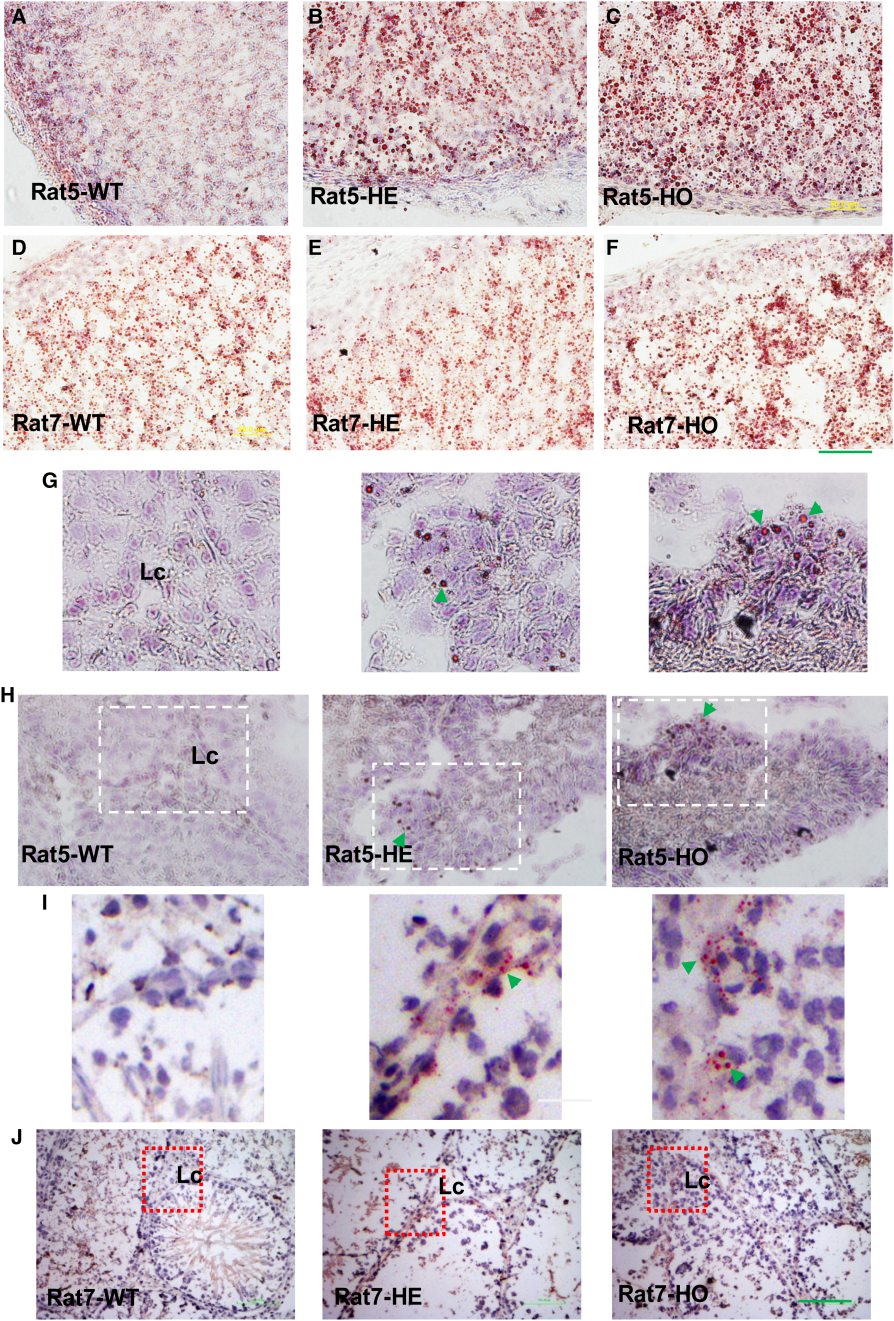
Effect of TSPO deletion on the accumulation of esterified cholesterol in rat models. (**A–J**) ORO staining of adrenal glands from WT, HE, and HO rats. HO rats exhibited increased ORO staining of neutral lipids, which reflected esterified cholesterol in the steroidogenic tissues. (**A–C**) Male adrenal gland of Rat5; scale bar = 50 µm. (**D–F**) Male adrenal gland of Rat7; scale bar = 50 µm. (**G–J**) The distribution of esterified cholesterol in Rat5 and Rat7 testis. Esterified cholesterol was estimated using ORO staining of testis from Rat5 (**G–H**) and Rat7 (**I–J**). WT, HE, and HO rats are indicated, and the highlighted area is magnified below each panel. Lc, Leydig cell. Green arrows indicate lipid droplets; scale bar = 100 µm.

**Figure 4.**
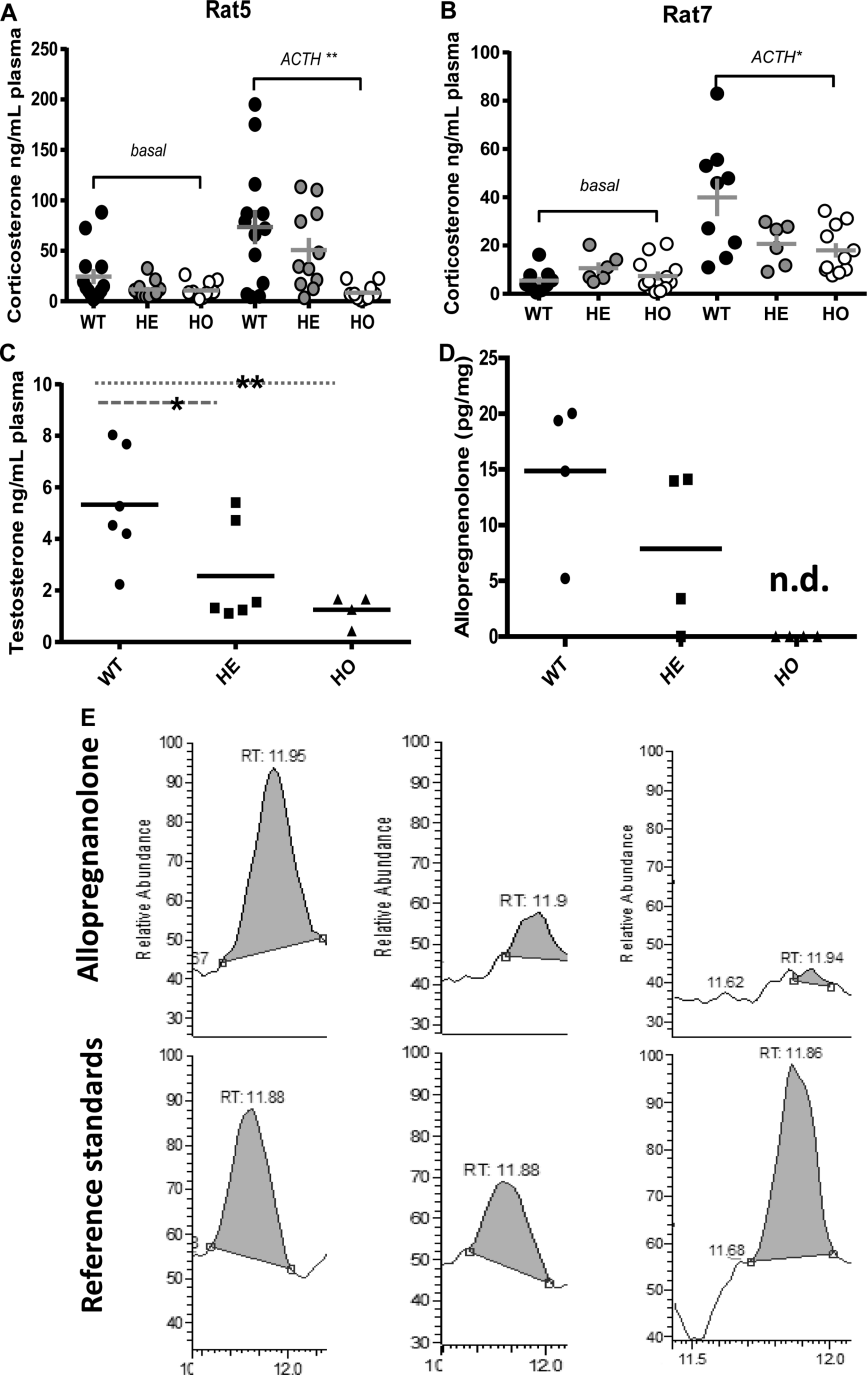
Measurement of steroid biosynthesis in rat models. (**A** and **B**) Circulating corticosterone levels in WT, HE, and HO Rat5 and Rat7 treated with and without ACTH. Plasma corticosterone levels from basal and ACTH-treated WT, HE, and HO Rat5 (**A**); Mann–Whitney *U-*test; **P < 0.01 (between ACTH-treated WT and HO; *n *= 10–13 animals per group). Plasma corticosterone levels from basal and ACTH-treated WT, HE, and HO Rat7 (**B**); Mann–Whitney *U-*test; *P *= 0.051 (between ACTH-treated WT and HO); *n *= 10–13 animals per group. (**C** and **D**) Circulating testosterone levels in Rat5 and allopregnanolone levels in Rat7 brains. Testosterone was measured using ELISA, and allopregnanolone was measured using LC–MS. (**C**) Basal testosterone levels in WT, HE, and HO from Rat5 (similar results were obtained in Rat7). Mann–Whitney *U-*test; *P < 0.05, **P < 0.01 (WT vs. HE or HO; *n *= 5–6 animals per group). (**D**) Relative amounts of allopregnanolone in Rat7 (WT, HE, and HO) cortex (not measurable in HO). n.d., not detectable; Student's *t*-test, *n *= 4. (**E**) Allopregnanolone in WT, HE, and HO Rat7 brains measured by UHPLC–QQQ MS. Representative chromatography of the allopregnanolone measurements from each Rat7 genotype: WT, HE, and HO. The reference standard is indicated.

Cholesterol-enriched lipid droplets in steroidogenic cells, the source of free cholesterol used for steroid formation, are visualized and quantified using ORO staining. Both Rat5 and Rat7 displayed increased the neutral lipid accumulation in both adrenal and testis as determined by ORO, indicating that there is a disturbance in free/esterified cholesterol homeostasis from inefficiently metabolized esterified cholesterol in the absence of intact TSPO (Figure 3A–J), possibly because free cholesterol was not used for steroid synthesis. Although there were no significant differences in basal circulating corticosterone levels between the WT, HE, and homozygous (HO) groups, the HO genotypes in both Rat5 and Rat7 failed/attenuated to produce corticosterone in response to ACTH treatment (Figure 4A,B). This could be attributed to mislocated cholesterol-binding and/or misfolded truncated protein (Supplementary Figure S4A–E). Similar accumulation of neutral lipids occurs in female adrenal glands, and other steroidogenic tissues such as ovary and testis, where the ORO staining in female tissues shows remarkable differences between the WT and HO (Figure 2I–L and Supplementary Figure S5A–L). Basal testosterone production and brain allopregnanolone levels were significantly reduced in Rat5 and Rat7, respectively (Figure 4C–E).

### Ala147/Thr substituation results in reduced affinity of TSPO for cholesterol

**Figure 5.**
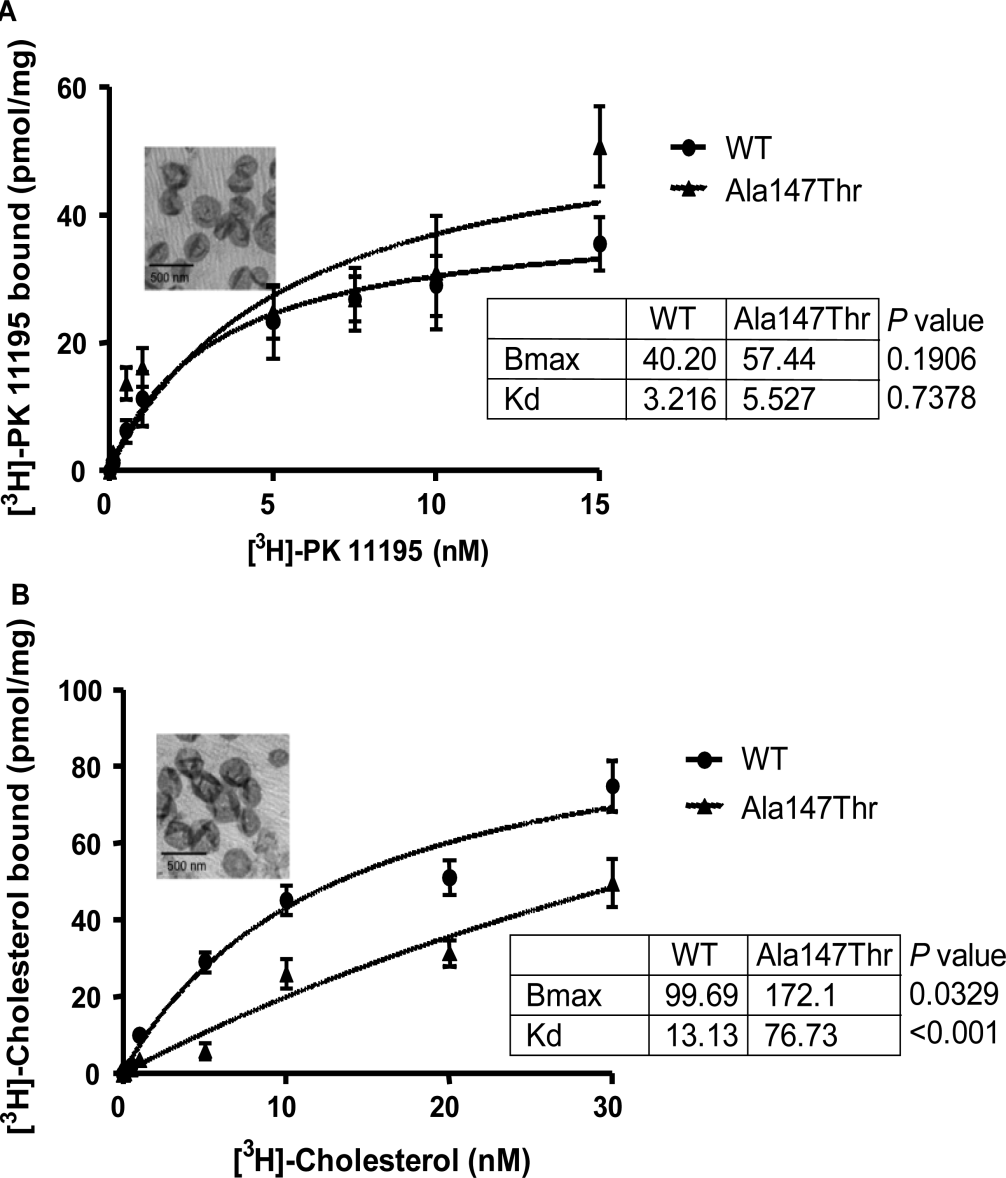
Effect of TSPO polymorphism on PK 11195/cholesterol binding *in vitro*. Saturation isotherms of [^3^H]-PK 11195 and [^3^H]-cholesterol binding to reconstituted mouse TSPO WT and mutant (Ala/Thr) proteins. (**A**) [^3^H]-PK 11195 and [^3^H]-cholesterol-specific binding studies were performed using 200 ng of mouse WT TSPO. Inset, electron micrographs of WT TSPO proteoliposomes stained with 2% uranyl acetate after SDS elimination using biobeads. (**B**) 200 ng of mouse Ala147Thr mutant reconstituted TSPO was used to study specific binding with [^3^H]-PK 11195 and [^3^H]-cholesterol. Inset, electron micrographs of mutant TSPO proteoliposomes stained with 2% uranyl acetate after SDS elimination using biobeads. [^3^H]-PK 11195 concentrations varied from 0.1 to 15 nM; [^3^H]-cholesterol concentrations varied from 0.1 to 30 nM. Values shown represent the mean (SE) from three independent experiments. The extra sum-of-squares *F-*test was used to compare the fitting curves and the *P-*values presented (values of *P* < 0.05 are statistically significant to reject the null hypothesis).

*In vitro* reconstitution experiments using recombinant rodent WT and mutant Ala147/Thr in proteoliposomes showed that both proteins, as predicted, bind the TSPO ligand [^3^H]-PK 11195 with similar affinities with no significant difference [24–26]. The mouse TSPO was used here because it is 96% homologous to the rat TSPO, and we have extensively studied this protein. The quality of the proeolipososmes produced using WT and mutant Ala147/Thr TSPO was the same as assessed by elctron microcopy (Figure 5A,B). However, the cholesterol affinity for Thr147 TSPO was six-fold lower than that for Ala147 TSPO, and the comparison of binding curve fittings is statistically significant (*P* < 0.05, *F-*test), indicating that the Ala147/Thr polymorphism affects cholesterol binding to mitochondrial TSPO and its import needed for steroid biosynthesis (Figure 5).

### Humans carrying rs6971 (Ala147/Thr) polymorphism show reduced response to ACTH treatment

**Figure 6.**
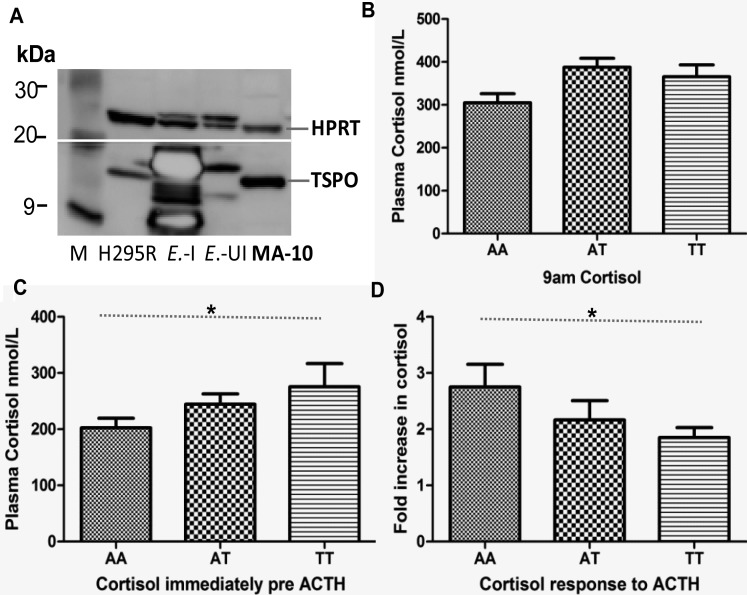
Humans carrying rs6971 (Ala147/Thr) polymorphism show reduced response to ACTH treatment. (**A**) Western blot analysis of human TSPO expression in the human steroid-producing adrenocortical cell line H295R. TSPO recombinant protein in *E. coli* (*E*.-I, induced with IPTG; *E*.-UI, uninduced cell lysate). The mouse Leydig cell line MA-10 was used as a positive control, and HPRT was used as a loading control. Biotinylated protein ladder marker sizes are labeled in kDa. (**B**) Plasma cortisol concentrations in healthy male volunteers at 09:00 AM, with AA (*n *= 18), AT (*n *= 16), and TT (*n *= 11) polymorphisms. A gene-dose effect was not observed. AA, 304.9 (18.12) nmol/l; AT, 387.8 (24.4) nmol/l; TT, 365.6 (27.80) nmol/l (*P *= 0.0629, linear trend test). Values shown represent the mean (SE). (**C**) Plasma cortisol concentrations immediately before ACTH administration (at 11:00 AM) in healthy male volunteers with AA (*n *= 20), AT (*n *= 16), and TT (*n *= 11) polymorphisms. A gene-dose effect was observed. AA, 202.7 (16.7) nmol/l; AT, 244.5 (22.6) nmol/l; TT, 275.4 (30.6) nmol/l (*P = 0.029, linear trend test). Values shown represent the mean (SE). (**D**) Fold-change in plasma cortisol concentration after ACTH administration (250 µg, IV). A gene-dose effect was observed (fold-change): AA (*n *= 10) 2.75 (0.32); AT (*n *= 10) 2.17 (0.25); TT (*n *= 10) 1.85 (0.22) (*P = 0.023, linear trend test). Values shown represent the mean (SE).

To assess the functional significance of the rs6971 (Ala147/Thr) polymorphism in humans, we analyzed TSPO protein expression in the well-established hormone-responsive steroid-producing human adrenocortical cell line H295R, and evaluated basal and ACTH-challenged cortisol production in healthy male volunteers [27]. H295R cells contain immunoreactive18 kDa TSPO protein (Figure 6A), confirming previous findings that these cells expressed *Tspo* mRNA that human adrenal cortex tissue contains high levels of immunoreactive TSPO shown by immunohistochemistry [28,29], and disproving a report that these cells do not express TSPO [5]. We observed that the increase in ACTH-induced plasma cortisol levels was genotype-dependent (Figure 6B–D). Subjects were genotyped at the rs6971 polymorphism as Ala147/Ala (AA), Ala147/Thr (AT), or Thr147/Thr (TT). We did not observe any genotype-related changes in the 09:00 AM plasma cortisol level (Figure 6B; *P *= 0.063, linear trend test). However, a gene-dose effect was observed in plasma cortisol level immediately before ACTH administration at 11:00 AM (TT > AT > AA; *P *= 0.029; Figure 6C). The ACTH-induced increase in plasma cortisol level also exhibited a gene-dose effect (AA > AT > TT; *P *= 0.023; Figure 6D). The mechanism mediating these results may be partly due to the reduction in TSPO–cholesterol-binding affinity, and thus steroid formation, as shown by both the *in vitro* reconstitution experiments using recombinant mutant Ala147/Thr TSPO and *in vivo* using the HO mutant Rat5 and Rat7 models.

## Discussion

An early and necessary step in steroid biosynthesis is the conversion of cholesterol into pregnenolone by CYP11A1 in mitochondria. This reaction depends on the delivery of cholesterol from intracellular stores to mitochondria. A wealth of evidence implicates the cholesterol-binding TSPO as a mediator in this delivery [30]. However, recent studies report that *Tspo* KO in mice performed by removing exons 2 and 3 of the gene does not affect steroid synthesis [4,5,7], although steroidogenic cell-targeted TSPO deletion abolished the corticosterone response to ACTH and affected lipid homeostasis in both adrenal and testis [6]. Although one should have expected that the removal of a gene or disruption or its expression, both resulting in lack of protein production, should have the same physiological effects, it seems that the responses are different and distinct compensation mechanisms are activated [31]. Thus, it is likely that mice can produce steroids in the absence of TSPO through an alternative pathway. The presence of such a mechanism is supported by the fact that TSPO is extremely conserved in nature and ubiquitously present across phylla and species, and there is extensive literature demonstrating its role in mitochondrial function of various tissues and steroid formation, an activity initiated in the mitochondria of steroidogenic cells.

Because of the discrepancies observed between various genetic models used to study TSPO in the mouse, e.g. gene deletion vs. exon removal, global gene deletion vs. tissue specific removal, we investigated the role of TSPO in the rat. Rat models have several advantages over mouse models and other organisms because of their well-characterized physiology and their extensive use in the development of novel therapeutic entities [32]. Using ZFN technology, we generated two *Tspo* global KO rat models, Rat5 and Rat7, both with TSPO deletion mutations, lacking the 18 kDa TSPO protein and the ability to bind the TSPO diagnostic ligand PK 11195 [33]. Our goal was to both disrupt TSPO expression (Rat5) and remove the fifth transmembrane loop region that includes the CRAC domain, including Ala147, responsible for cholesterol binding (Rat7) [9]. Rat5 and Rat7 did not display significantly aberrant phenotypes, suggesting that a compensatory mechanism may have replaced some of the functions of TSPO in these two lines.

Cholesterol for steroidogenesis is stored in the cholesteryl ester-enriched lipid droplets in steroidogenic cells [34,35]. Sustainable steroidogenesis depends on the availability of intracellular free cholesterol, a major part of which is provided by de-esterification of cholesterol esters in lipid droplets. Thus, in steroid-synthesizing cells, the levels of esterified cholesterol are an important morphological characteristic reflecting the use of free cholesterol for steroid biosynthesis [36–39]. Both rat lines carrying a *Tspo* deletion mutation displayed increased neutral lipid accumulation in adrenal, ovary, and testis as determined by ORO staining, indicating that esterified cholesterol was inefficiently metabolized in the absence of intact TSPO. In agreement with the increased neutral lipid accumulation, in both rat lines, the response to ACTH was absent or attenuated in homozygotes. Similar findings, increased neutral lipid accumulation coupled with impaired ACTH-induced steroid production, were reported following the targeted disruption of aldosterone synthase, STAR, CYP11A1, and TSPO in mice [6,40–42]. In the case of TSPO, the disturbance of lipid homeostasis was reported in a *Tspo* conditional KO mouse model created using adrenal gland and/or testis-specific *Nr5a1*-Cre mouse line [6].

Although the two TSPO mutant rat lines showed a non-detectable phenotype, in addition to the lack of a response to ACTH, we observed reduced circulating testosterone levels and undetectable allopregnanolone in the brain cortex. The reduced testosterone levels were to be expected, because we also noted increased ORO staining in testis. We were unable to detect the neurosteroid allopregnanolone in Rat7 brain cortex. Although this was predictable given the role of TSPO in neurosteroid formation, we would have expected a neurological/behavioral phenotype to accompany this reduction considering the reported role of allopregnanolone in development and brain function [43,44]. However, at present, we have not undertaken behavioral tests, and we have not exposed the animals to various stressors. Moreover, we have not examined other areas of the brain for their ability to form allopregnanolone or other neurosteroids.

*In vitro* reconstitution experiments showed a significantly reduced cholesterol affinity for Thr147 relative to that of Ala147 recombinant mouse TSPO, suggesting that altered cholesterol affinity, and thus reduced mitochondrial cholesterol import, may mediate the effects on cortisol production. Here, we demonstrated that the fifth transmembrane loop of TSPO, that includes Ala 147 and the CRAC domain, is critical for ACTH-induced corticosteroid formation in the rat. Consistent with this, healthy male volunteers carrying the rare allele of the rs6971 polymorphism that affects the fifth transmembrane loop (Thr147 TSPO) [10] displayed a reduced plasma cortisol rate of formation in response to ACTH challenge compared with healthy volunteers carrying the common allele (Ala147). Furthermore, heterozygote volunteers had an intermediate response implying a gene-dose effect. In human, Ala147/Thr polymorphism within the fifth transmembrane loop disrupts hormonal regulation of corticosteroid synthesis, which may be partly explained by the reduction in cholesterol-binding affinity for Thr147 TSPO. These data are supported by recent structural studies on TSPO from *Rhodobacter sphaeroides* [45].

Taken together, the results presented here support the role of TSPO in hormone-stimulated steroid biosynthesis and the role of TSPO ligands in diseases with steroid-dependent stress and anxiety components [2,46,47]. Further experimental studies on the TSPO ligand-binding domain(s) will advance therapeutic drug discovery for human diseases associated with altered steroid and neurosteroid synthesis [1,2].

## Abbreviations

ACTH: adrenocorticotropic hormone
CRAC: cholesterol recognition amino acid consensus
ELISA: enzyme-linked immunosorbent assay
hCG: human chorionic gonadotropin
HE: heterozygote
IF: immunofluorescence
KO: knockout
ORO: Oil Red O
PFA: paraformaldehyde
QQQ: triple quadrupole mass spectrometry
TSPO: translocator protein
UHPLC: ultrahigh performance liquid chromatography
WT: wild type
ZFN: zinc finger nuclease
