# Supplementary material for: TSPO mutations in rats and a human polymorphism impair the rate of steroid synthesis
Source: Biochem J. 2017 Nov 21;474(23):3985–99. doi: 10.1042/BCJ20170648 (PMC5697202; doi:10.1042/BCJ20170648)
Supplement: Supplementary Figures [file BCJ-474-3985-s1.pdf]

Supplementary Material

Figure S1. Genome editing of the rat *Tspo* locus using optimized and customized ZFN technology and RT-PCR of Rat5 and Rat7 founders.

(A and B) Modifications were made to the ZFN of the *Tspo* gene in the rat C6 cell line for optimized (A) and customized (B) genome targeting: GGCATGGCCCCCCATCTTctttggTGCCCGGCAGATGGGCTG, and GGCCTTTGCCACCATGCTcaactACTATGTATGGCGTGATAAC, respectively. Images were collected from the Cel-1 assay of the digested products resolved on 10% TBE-PAGE using the Surveyor Mutation Detection assay. Neg, control cells; DNA, cells transfected with plasmids; RNA, cells transfected with mRNAs. WT size is indicated as 367 bp for optimized (A) and 316 bp for customized (B) genome editing.

(C) Chromatography of the mutated region in Rat5 *Tspo* mRNA. RT-PCR was used to show deletion and intron insertions in mutant mRNA. Deleted sequence and introduced intron sequence are indicated. The partial untouched exon 4 is indicated.

(D) Chromatography of the mutated region in Rat7 *Tspo* mRNA. RT-PCR was used to show DNA repair, deletion, and insertion in the mRNA (red arrows). Deleted sequence leading to a new stop codon (TAG) is indicated.

Figure S2. Predicted Rat5 and Rat7 TSPO mutations and wild-type TSPO.

(A) Sequence alignments of the predicted Rat5 and Rat7 TSPO mutations and WT TSPO. The predicted new protein was based on the sequences of the mutant Rat5 and Rat7 *Tspo* mRNAs. The new C-termini without the CRAC motif are indicated. The theoretical pI/MW (isoelectric point/molecular weight) of the expected Rat7 TSPO and WT TSPO are given as follows: 6.53/14026.29 (Rat5), 9.21/16488.25(Rat7), and 9.69/18940.02 (WT).

(B) Transmembrane domain profiles of predicted Rat5 and Rat7 TSPO mutations and WT TSPO proteins. TM1-5, transmembrane domain 1 to 5. The graphs were created using the TMHMM Server v. 2.0 (http://www.cbs.dtu.dk/services/TMHMM).

Figure S3. PK 11195 binding assay using adrenal glands from wild-type, Rat5, and Rat7 females.

(**A and C)** Representative optical bright-field images were used as controls to show the adrenal section morphologies. **B and D.** Autoradiographic localization of PK 11195 in the adrenal glands from WT, Rat5, and Rat7 females incubated with 1.2 nmol/L [^{3}H]-PK 11195 in 50 mmol/L Tris-HCl pH 7.4 (B), or incubated with 10 μM cold PK 11195 to determine non-specific binding (D). The [^{3}H]-PK 11195 binding was analyzed using digital autoradiography with the Beta-Imager 2000 (Biospace Lab, Paris, France). Two representative animals from each group [WT, heterozygotes (HE) and homozygotes (HO)] were used for the analysis.

Figure S4. Homology models and small molecule docking of rat wild-type TSPO and Rat7 mutated TSPO.

(A) Homology model of rat TSPO based on the template NMR structure of mouse TSPO. The CRAC motif and N-terminal (N-) are indicated.

(B) Homology model of Rat7 mutated TSPO based on the template NMR structure of mouse TSPO. The new C-terminus without the CRAC motif is indicated.

(C and D) Rat TSPO docking with cholesterol. Protein and small molecule docking were performed using whole protein as the docking grid box. The cholesterol was docked to the rat TSPO model at its C-terminal CRAC motif with high affinity. CRAC and cholesterol (Chol) are indicated. The five transmembrane domains of rat TSPO are shown as a yellow surface in D, where the –OH group of cholesterol is exposed on the outer mitochondrial membrane.

(E) Rat7 mutated TSPO docking with cholesterol. The Rat7 mutated TSPO without the CRAC motif appears to disrupt cholesterol binding to its C-terminus. The cholesterol was predicted to bind a potential hydrophobic pocket with an affinity of –8.5 kCa/mol. This mislocalization of cholesterol in Rat7 TSPO may affect the protein's use of the substrate for stimulated adrenal steroid biosynthesis.

Figure S5. Distribution of esterified cholesterol in adrenal glands and ovaries of Rat5 and Rat7 females.

(A–F) The female Rat5 HO exhibited significantly increased Oil Red O staining of neutral lipids, which reflects esterified cholesterol accumulation in steroidogenic tissues: adrenal glands (A–C) and ovaries (D–F).

(G–L) The female Rat7 HO exhibited significantly increased esterified cholesterol accumulation in adrenal glands (G–I) and ovaries (J–L), which is similar to that observed in Rat5 HO. The ID of each representative rat is indicated (*e.g.*, WT2384, HE2356, HO2355). Scale bar=50 μm.

Fig. S1

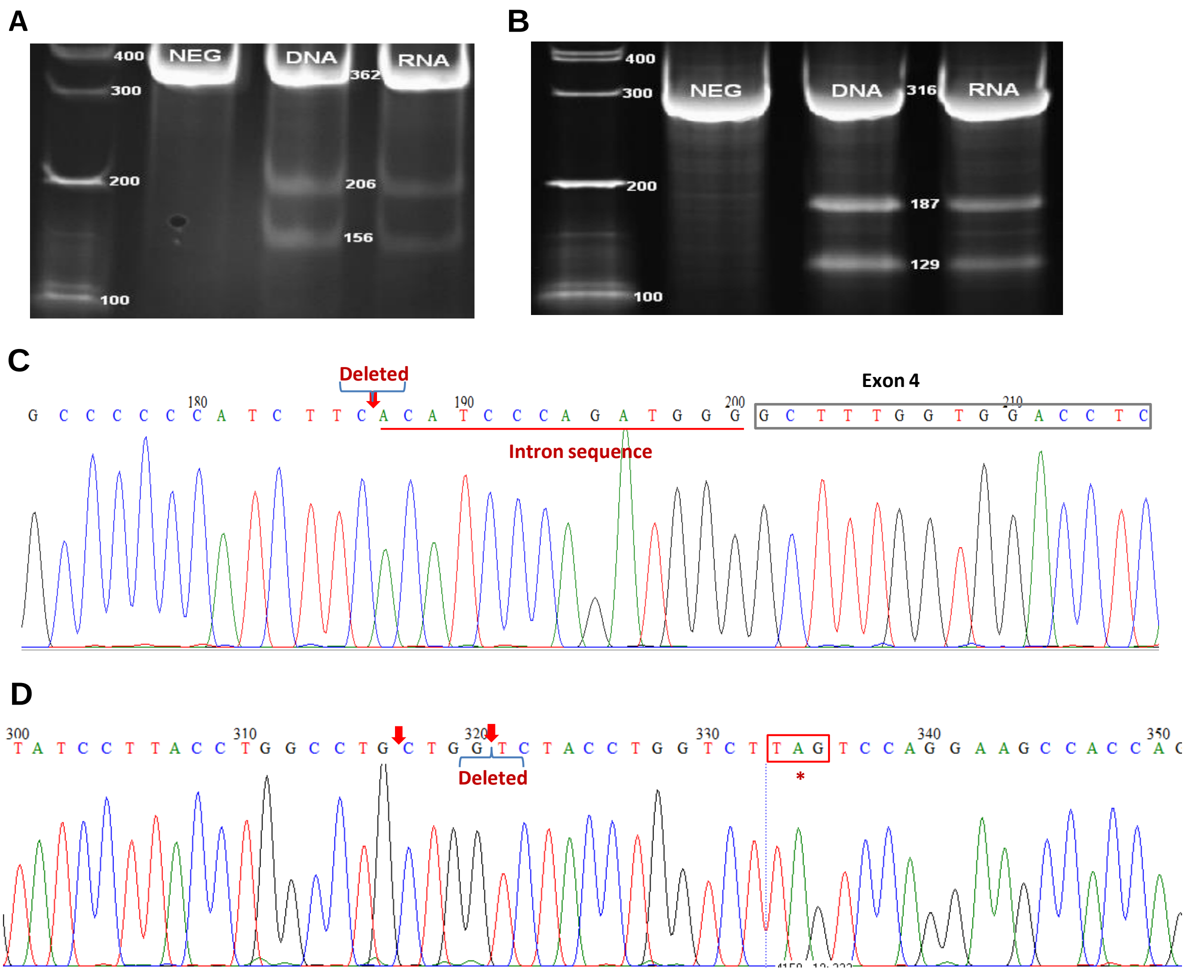
A
400
300
200
100
NEG
DNA
RNA
362
206
156
B
400
300
200
100
NEG
DNA
RNA
316
187
129
C
Deleted
Exon 4
180
190
200
210
G C C C C C C A T C T T C A C A T C C C A G A T G G G G C T T T G G T G G A C C T C
Intron sequence
D
300
310
320
330
340
350
T A T C C T T A C C T G G C C T G C T G G T C T A C C T G G T C T T A G T C C A G G A A G C C A C C A C
Deleted
*

Fig. S2

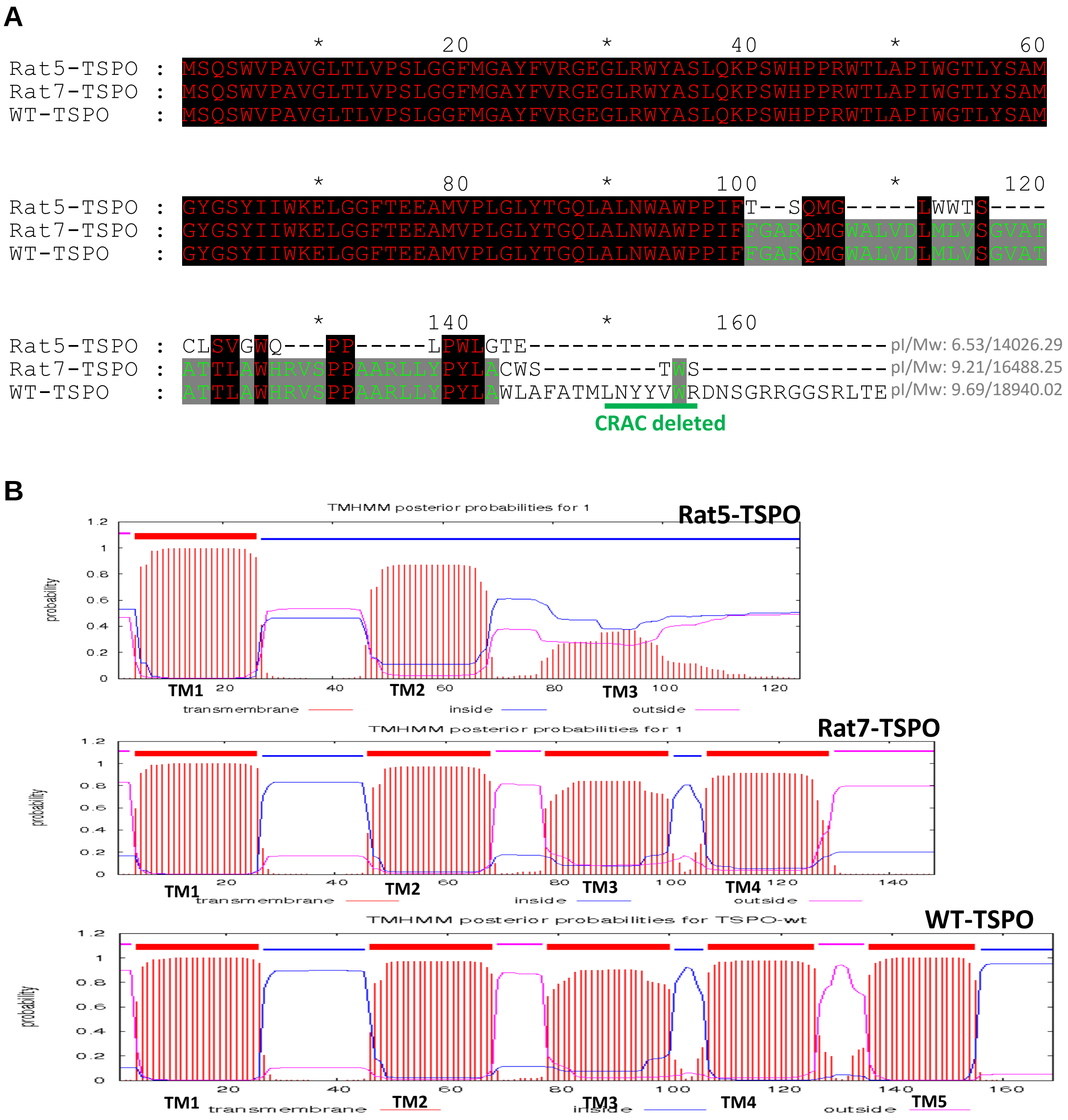
A
* 20 * 40 * 60
Rat5-TSPO : MSQSWVPAVGLTLVPSLGGFMGAYFVRGEGLRWYASLQKPSWHPPRWTLAPIWGTLYSAM
Rat7-TSPO : MSQSWVPAVGLTLVPSLGGFMGAYFVRGEGLRWYASLQKPSWHPPRWTLAPIWGTLYSAM
WT-TSPO : MSQSWVPAVGLTLVPSLGGFMGAYFVRGEGLRWYASLQKPSWHPPRWTLAPIWGTLYSAM
* 80 * 100 * 120
Rat5-TSPO : GYGSYIIWKELGGFTEEAMVPLGLYTGQLALNWAWPPIFT--SQMG-----LWWTS----
Rat7-TSPO : GYGSYIIWKELGGFTEEAMVPLGLYTGQLALNWAWPPIFFGARQMGWALVDLMLVSGVAT
WT-TSPO : GYGSYIIWKELGGFTEEAMVPLGLYTGQLALNWAWPPIFFGARQMGWALVDLMLVSGVAT
* 140 * 160
Rat5-TSPO : CLSVGWQ---PP-----LPWLGTE-------------------------- pI/Mw: 6.53/14026.29
Rat7-TSPO : ATTLAWHRVSPPAARLLYPYLACWS--------TWS-------------- pI/Mw: 9.21/16488.25
WT-TSPO : ATTLAWHRVSPPAARLLYPYLAWLAFATMLNYYVWRDNSGRRGGSRLTE pI/Mw: 9.69/18940.02
CRAC deleted
B
TMHMM posterior probabilities for 1
Rat5-TSPO
probability
TM1 TM2 TM3
transmembrane inside outside
TMHMM posterior probabilities for 1
Rat7-TSPO
TM1 TM2 TM3 TM4
transmembrane inside outside
TMHMM posterior probabilities for TSPO-wt
WT-TSPO
TM1 TM2 TM3 TM4 TM5
transmembrane inside outside

Fig. S3

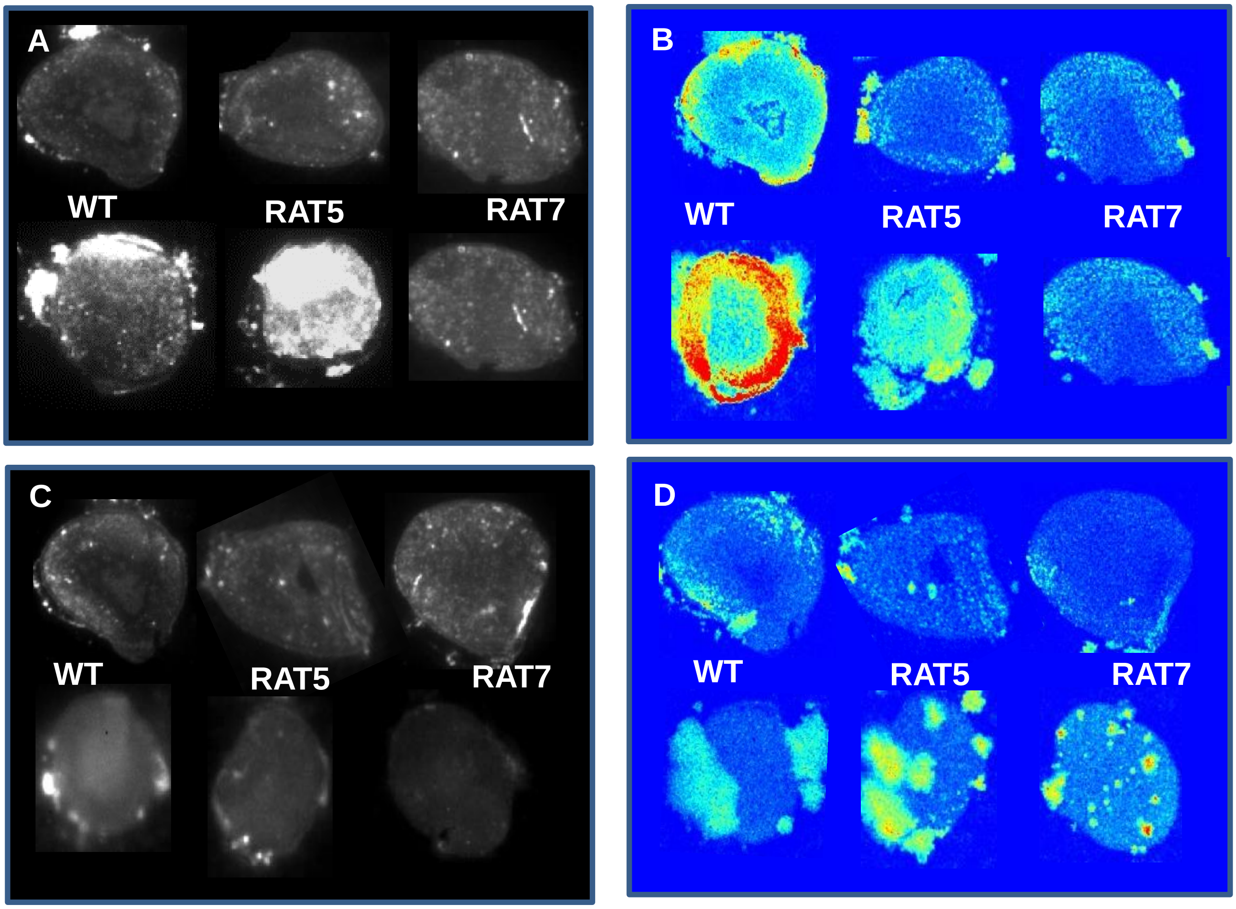

Fig. S4

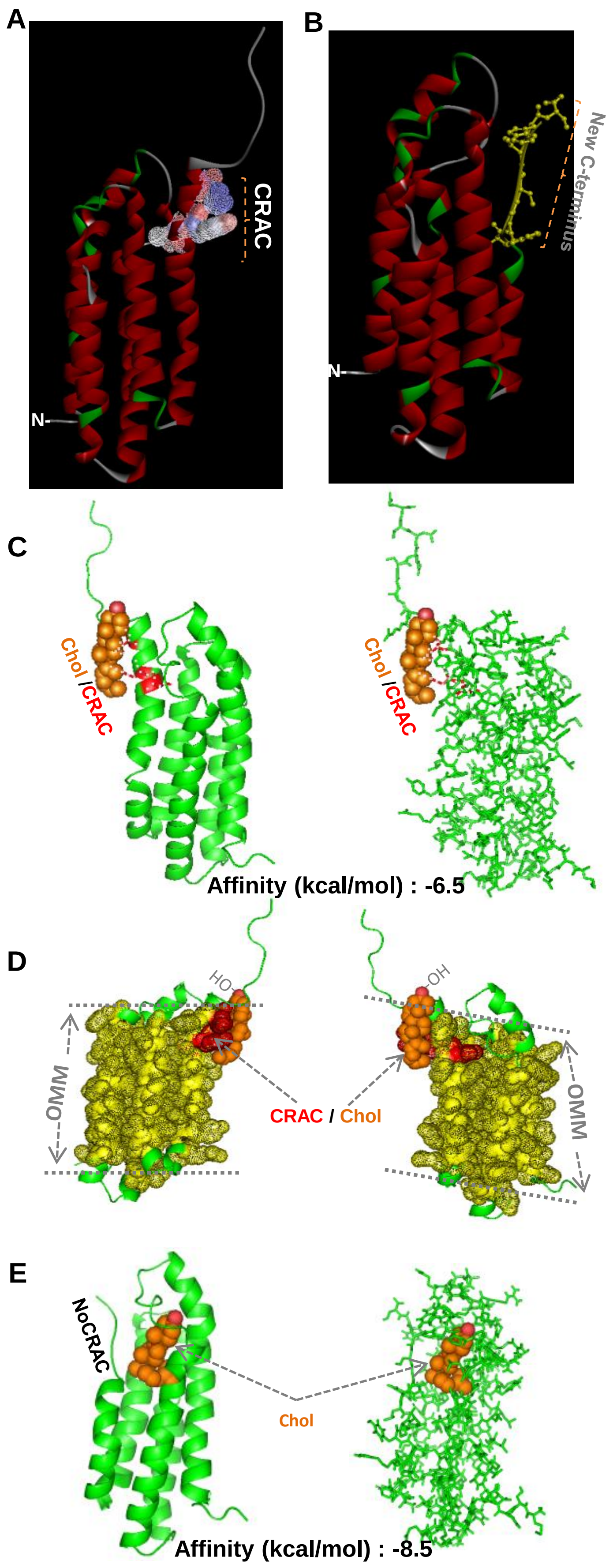
A
CRAC
N-
B
New C-terminus
N-
C
Chol /CRAC
Chol /CRAC
Affinity (kcal/mol) : -6.5
D
HO
OH
OMM
CRAC / Chol
OMM
E
NoCRAC
Chol
Affinity (kcal/mol) : -8.5

Fig. S5

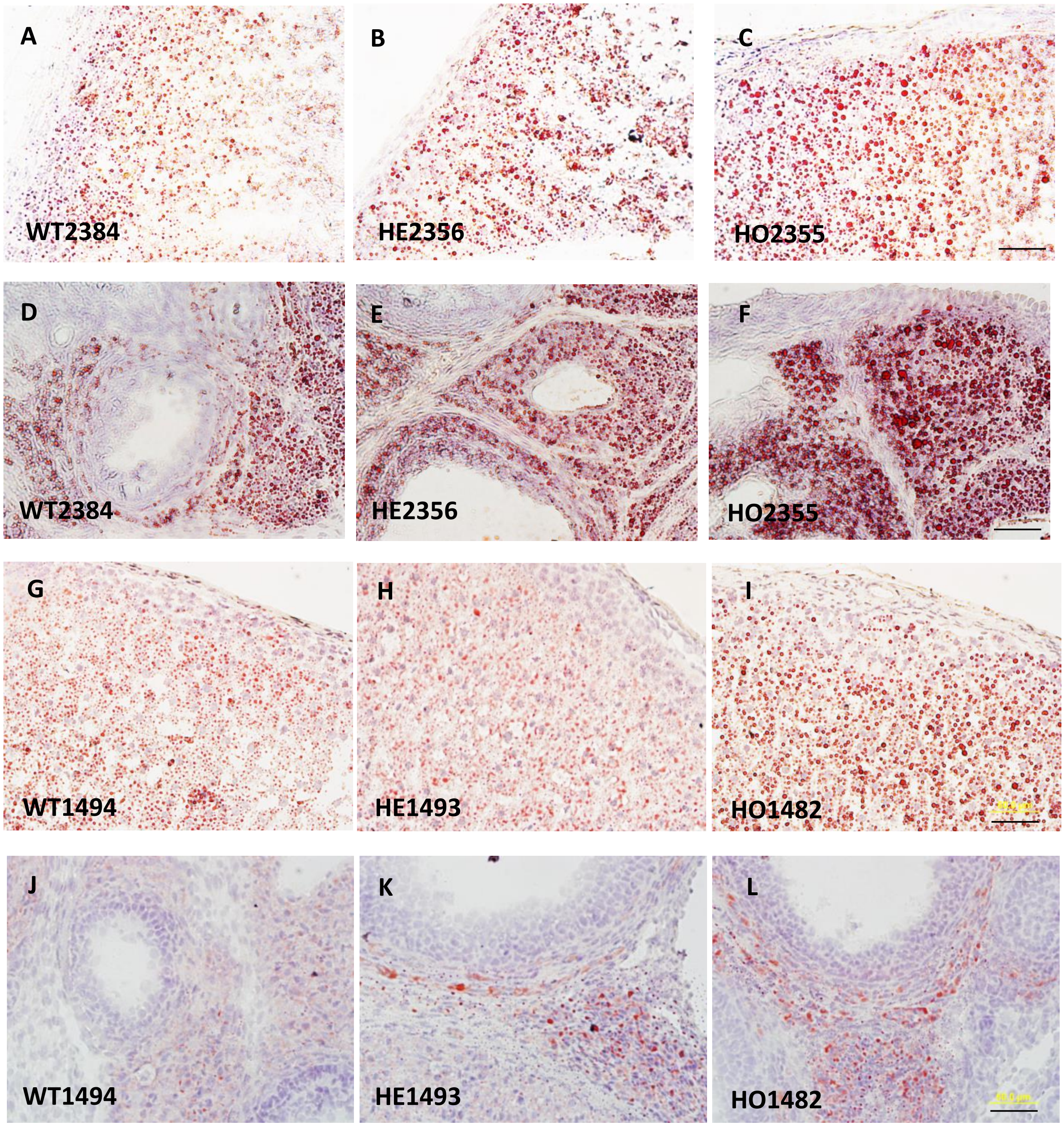
A
WT2384
B
HE2356
C
HO2355
D
WT2384
E
HE2356
F
HO2355
G
WT1494
H
HE1493
I
HO1482
J
WT1494
K
HE1493
L
HO1482
